# An Automated Cell-Free Workflow for Transcription Factor Engineering

**DOI:** 10.1021/acssynbio.4c00471

**Published:** 2024-10-07

**Authors:** Holly M. Ekas, Brenda Wang, Adam D. Silverman, Julius B. Lucks, Ashty S. Karim, Michael C. Jewett

**Affiliations:** † Department of Chemical and Biological Engineering, Northwestern University, Evanston, Illinois 60208, United States; ‡ Chemistry of Life Processes Institute, Northwestern University, Evanston, Illinois 60208, United States; § Center for Synthetic Biology, Northwestern University, Evanston, Illinois 60208, United States; ∥ Center for Engineering Sustainability and Resilience, Northwestern University, Evanston, Illinois 60208, United States; ⊥ Robert H. Lurie Comprehensive Cancer Center, Northwestern University, Chicago, Illinois 60611, United States; # Simpson Querrey Institute, Northwestern University, Chicago, Illinois 60611, United States; ¶ Department of Bioengineering, Stanford University, Stanford, California 94305, United States

**Keywords:** cell-free gene expression, synthetic biology, transcription factor, high-throughput, robotic liquid handling, protein engineering

## Abstract

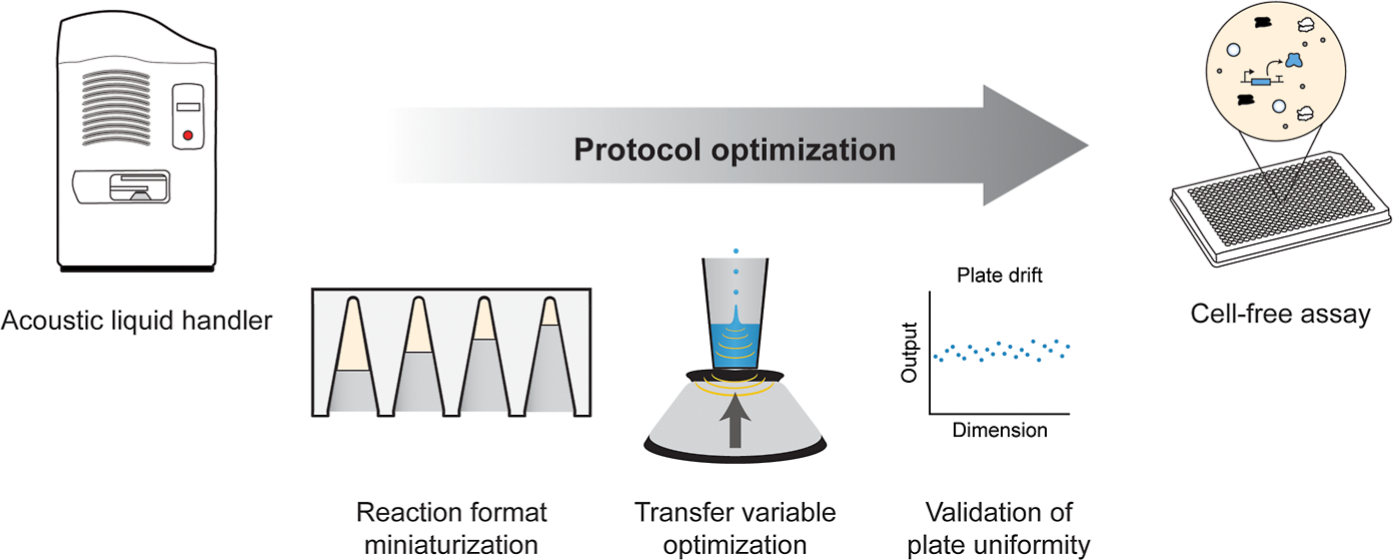

The design and optimization of metabolic pathways, genetic systems, and engineered proteins rely on high-throughput assays to streamline design-build-test-learn cycles. However, assay development is a time-consuming and laborious process. Here, we create a generalizable approach for the tailored optimization of automated cell-free gene expression (CFE)-based workflows, which offers distinct advantages over in vivo assays in reaction flexibility, control, and time to data. Centered around designing highly accurate and precise transfers on the Echo Acoustic Liquid Handler, we introduce pilot assays and validation strategies for each stage of protocol development. We then demonstrate the efficacy of our platform by engineering transcription factor-based biosensors. As a model, we rapidly generate and assay libraries of 127 MerR and 134 CadR transcription factor variants in 3682 unique CFE reactions in less than 48 h to improve limit of detection, selectivity, and dynamic range for mercury and cadmium detection. This was achieved by assessing a panel of ligand conditions for sensitivity (to 0.1, 1, 10 μM Hg and 0, 1, 10, 100 μM Cd for MerR and CadR, respectively) and selectivity (against Ag, As, Cd, Co, Cu, Hg, Ni, Pb, and Zn). We anticipate that our Echo-based, cell-free approach can be used to accelerate multiple design workflows in synthetic biology.

## Introduction

Engineering biological processes often requires the time-consuming construction of hundreds to thousands of unique cell lines, each with a single genetically encoded design of a protein, circuit, or biosynthetic pathway.^1^ To address this challenge, cell-free gene expression (CFE) systems have matured to enable the rapid testing of large combinations of biological functions via high-throughput screens.^2−4^ CFE systems work by combining crude cellular extracts capable of transcription and translation, reaction components (e.g., energy sources, cofactors, and salts), and DNA encoding the protein or genetic system to be expressed.^5−7^ CFE screens take advantage of several features of in vitro systems, including their open reaction environment, scalability, and time to data.^8−23^ In addition, laborious and time-consuming plasmid amplification methods (transformation and purification) can be circumvented through the polymerase chain reaction (PCR) amplification of linear expression templates (LETs) encoding the promoter, gene, and terminator.^9,24−28^ Furthermore, many parts of CFE reaction assembly can be automated with speeds and volumes inaccessible by manual setup using liquid-handling robots such as the Echo Acoustic Liquid Handler (Echo).^16,29^

Numerous studies have taken advantage of Echo-based high-throughput, cell-free screens. For example, a combinatorial space of ∼4,000,000 cell-free buffer compositions was studied for maximizing protein production.^16^ CFE systems have also been used in other high-throughput screens, such as creating a self-driving autonomous machine to accelerate engineering of glycoside hydrolase enzymes with enhanced thermal tolerance,^30^ discovering antibody sequences,^9^ or engineering biosensors.^23^ Key features of high-throughput, CFE screens are the precise control over reaction setup, the ability to manipulate multiple variables at once, and the granularity of the data set. Unfortunately, such assay parameters are often carried out in an ad hoc way without clearly defined validation protocols that would be necessary to build from or repeat the work. As a result, poor consistency and measurable, repeatable instances of drift with Echo protocols have been documented.^31−33^

**Figure 1:**
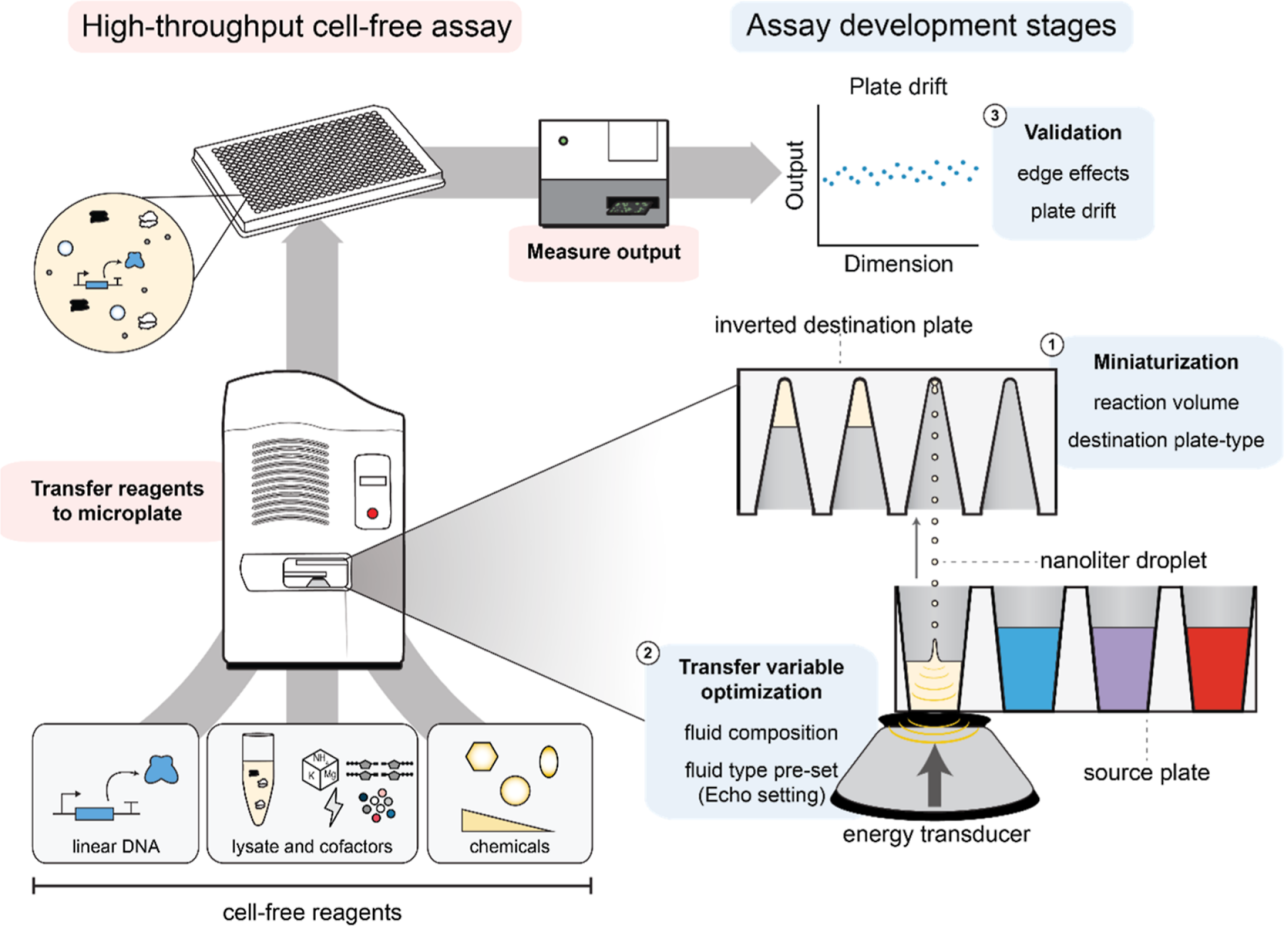
Robotic liquid handling enables the efficient setup of miniaturized cell-free reactions. Schematic describing an Echo-based assay workflow. The Echo Acoustic Liquid Handler uses acoustic energy to transfer nanoliter-sized droplets from source plates to destination plates. Miniaturized CFE reactions can be constructed by combining nanoliter-volume transfers of cell-free reaction components in plates. Then, CFE reactions can be incubated or read directly on a plate reader. Assay workflows are individualized to the application, requiring the optimization of (i) final reaction format, (ii) transfer variables, and (iii) validation via pilot assays.

With an eye toward facilitating access to, and understanding of, high-throughput CFE assays for massively parallelized combinatorial reactions, we develop a general workflow for CFE parameter validation when using Echo robots (Figure 1). Our workflow involves three steps that assess the final reaction format and volume, fluid transfer (e.g., fluid consistency, viscosity, and fluid tension), and plate uniformity. We find that these critical parameters can be tuned for improved consistency, precision, and confidence. To demonstrate the efficacy of this validation workflow, we engineer two allosteric transcription factor (aTF) biosensors by calculating fold-change to measure whole-library limit of detection shifts spanning 100-fold concentration difference, as well as selectivity preferences against a panel of 9 ligands. In total, we assembled 7364 individual 1 μL CFE reactions (including controls and two replicates) in 48 h with high precision, matching the data accuracy of manually assembled reactions.

## Results and Discussion

The goal of this work was to develop validation parameters for implementing high-throughput CFE workflows using the Echo. To assemble CFE reactions, the Echo individually transfers nanoliter-volume reagents between a source and destination plate (Figure 1). The operator programs the Echo with the source and destination well-coordinates, expected fluid type, and desired transfer volume for each unique reagent transfer. Once all transfers are completed, the destination plate could be incubated or read directly. Ideally, Echo assay workflow protocols are individualized and optimized for their application. Unique fluid properties, such as surface tension and viscosity, will vary between reagents and affect transfer quality.^34^ We focused on (i) miniaturization (i.e., reaction volume and destination plate type), (ii) fluid transfer (i.e., fluid composition and fluid pretype set), and (iii) plate effects (i.e., edge effects and drift) (Figure 1).

### Signal Accuracy and Range When Dispensing at Small Volumes

**Figure 2:**
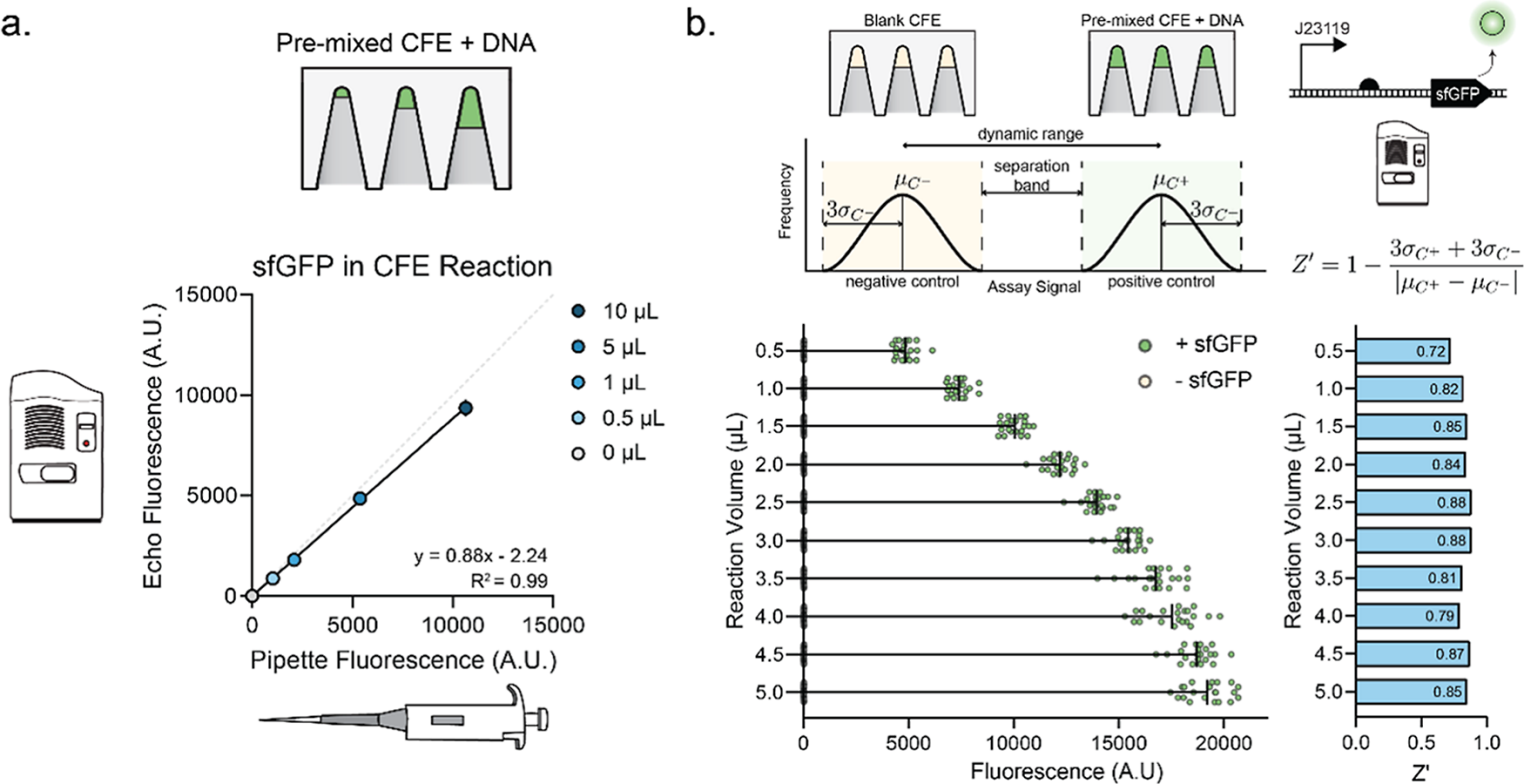
Miniaturization of cell-free reactions. (A) Echo transfer volume correlates with pipet volume standard. J23119-sfGFP DNA was combined into a single mixture and either transferred using the Echo or pipetted by hand for 0, 0.5, 1, 5, and 10 μL reaction. Plotted data represents average with standard deviation of three technical replicates (*n* = 3). Solid black line represents simple linear regression with equation and *R*^2^ shown on graph. Gray dotted line represents line of identity. (B) Schematic design and data for *Z*′ factor assay. Positive and negative assay controls, defined as CFE with and without J23119-sfGFP DNA, are selected to represent ideal assay range. *Z*′ factor is calculated as shown from the average and standard deviation of the controls, representing dynamic range and separation between signals. (Left graph) positive and negative controls were dispensed using the Echo 525 at volumes spanning 0.5–5 μL in 0.5 μL increments. Plotted data represent each experimental replicate. Ten reactions for each condition were set up on two separate days and plotted together. (Right graph) *Z*′ factor was calculated using the provided equation for all volumes. The *Z*′ factor is displayed on each bar, with 0.5 μL displaying the lowest *Z*′ factor. *Z*′ > 0.5 is acceptable for high-throughput screens.

We first set out to determine the minimum assay volume that would yield consistent and significant signal activation for plate-based CFE reactions using the Echo. Small reaction volumes (e.g., nL- to single μL-volumes) are desirable because they can be assembled more rapidly^13,35^ and reduce overall reagent costs. To assess dispense accuracy, we preassembled a CFE reaction mix expressing superfolder green fluorescent protein (sfGFP) via a highly active bacterial promoter, J23119,^36^ and dispensed this mix into individual reactions at 0, 0.5, 1, 5, and 10 μL manually (hand pipetted) and by the Echo (automated). Measurements from manual- and Echo-dispensed reactions showed a linear correlation across reaction volume (*R*^2^ = 0.99; Figure 2A), as has been previously observed.^37^ However, Echo transferred reactions produced a lower fluorescence compared with manual transfer for each volume, which could be due to a miscalibration of the Echo transfer.

We next assessed whether smaller reaction volumes led to a reduction in the assay signal range affecting our ability to discern signal over noise. We used *Z*′-factor—a function of the mean and standard deviation—as a measurement of our assay’s ability to identify significant activity above inherent data noise (Figure 2B).^11,38^ We again dispensed CFE premixed with either DNA or water using the Echo at volumes ranging from 0.5 to 5 μL in 0.5 μL increments (Figure 2B). At all volumes, our reactions that included template show distinct separation from the negative control. The average *Z*′ was 0.83 with a standard deviation of 0.05 across both days. *Z*′ close to one is considered ideal, representing a large dynamic range and separation band between the data, while *Z*′ greater than 0.5 is defined as sufficient for high-throughput screens.^38^ All *Z*′ were greater than 0.5. Furthermore, all *Z*′ are within one standard deviation of the average except for 0.5 μL, the smallest volume. Based on these data, we concluded that the Echo transfer accuracy is sufficient, and 1 μL reaction volumes are ideal for the purposes of dispensing CFE reactions.

### Fluid Composition and Setting Optimization for Small Volumes

**Figure 3:**
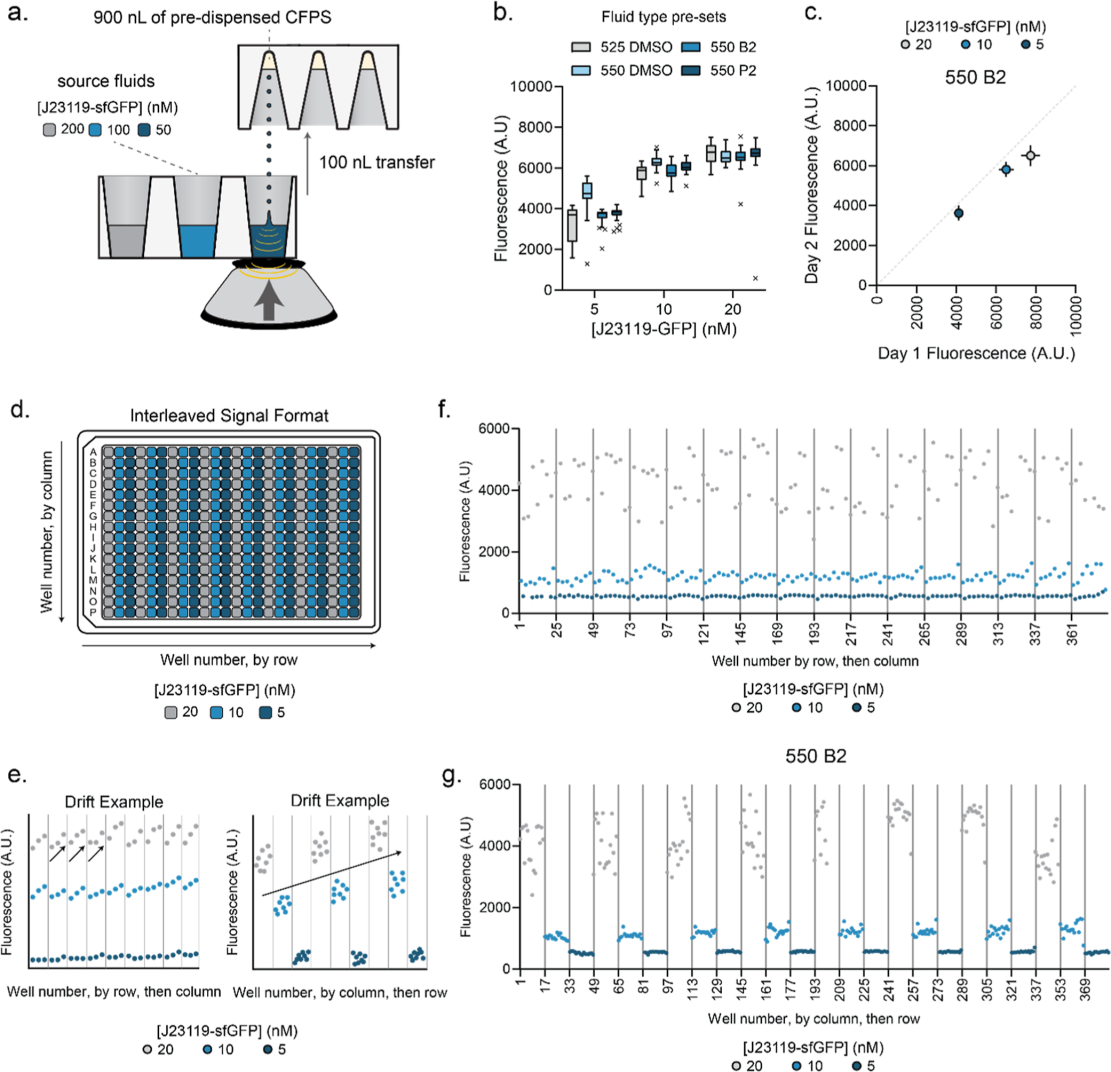
Optimization of DNA library transfer variables. (A) Schematic showing Echo transfer variable optimization for a 100 nL transfer of DNA. 100 nL of J23119-DNA at 200, 100, and 50 nM is transferred to 1 μL of CFE to yield ∼20, 10, and 5 nM final concentration of DNA in reaction. Different concentrations are used in subsequent precision assays to represent the concentration dependence of the allosteric transcription factor library. (B) Comparison of fluid type presets for 100 nL transfers on both Echo 525 and Echo 550. Reactions are graphed with the Tukey method. Data represent 32 technical replicates for each concentration/preset pair. (C) Echo 550 B2 preset shows consistent precision and accuracy when dispensing 100 nL of DNA between 2 days. Points represent average, and error bars show standard deviation of 32 technical replicates. Dotted gray line is line of identity. (D) Schematic of interleaved plate assay to identify drift and edge effects. 20, 10, and 5 nM J23119-sfGFP reactions are set up in alternating columns on a plate as a pilot assay. (E) Examples of column and row drift from interleaved plate assay. Trends from left to right or top to bottom can indicate drift. Differences between trends in the middle of the plate and edges can also indicate drift. (F) Cell-free pilot assay results graphing well number by row, then by column. Data points depict individual 1 μL of CFE reactions dispensed via Echo with 100 nL 200, 100, and 50 nM J23119-sfGFP dispensed in alternating columns using the 550 B2 setting. Gray dots represent 20 nM, blue represent 10 nM, and dark blue represent 5 nM. Gray lines denote groupings of rows. No material drift or edge effects were seen. (G) Cell-free pilot assay results graphing well number by column, then by row. Data points are the same as seen in (F) but graphed differently. Gray lines denote groupings of columns. No material drift or edge effects were observed.

After identifying an accurate and reliable reaction size, we sought to optimize the fluid transfer parameters for assembling Echo-based CFE reactions. Selecting optimal transfer settings (e.g., fluid composition and type) leads to higher accuracy and lower error during the reaction assembly. To optimize these transfers, we evaluated individual CFE reagents starting with DNA solutions. DNA in typical PCR buffers is well tolerated in CFE (Figure S1) and offers a controllable variable capable of tuning reaction output (i.e., fluorescence; Figure S2). We chose to transfer 100 nL of DNA, or ∼10% of the CFE reaction volume, as it was sufficient for delivering the necessary amount of DNA to our reaction. Therefore, we aimed to identify fluid parameters that would maximize CFE reaction precision from a 100 nL transfer of DNA at 5, 10, and 20 nM final concentrations of J23119-GFP (Figure 3A). To do this, 100 nL of source DNA at 50, 100, or 200 nM prepared in 100% PCR buffer was transferred to 900 nL of CFE reaction. Transfers were accomplished using one of two Echo models: the 525 that transfers in increments of 25 nL droplets or the 550 that transfers in increments of 2.5 nL droplets. For both models, droplet size and consistency can be controlled by the operator by selecting a plate type^39^ and a predefined fluid setting that approximates the reagent fluid properties to improve calculations of how much power the Echo needs to create droplets of the defined size (a preset parameter).^40^ Using the Echo-qualified low dead volume (LDV) 384-well plate, we assessed 100 nL DNA solution transfers with the DMSO (expected 70–100% v/v DMSO) preset available on both 525 and 550 as well as B2 (simple buffers without protein) and P2 (buffers or nonsurfactant buffers with proteins) presets available on the 550 (Figure 3B). The 525 had the largest interquartile range, likely due to a low number of 25 nL droplets compared to a higher number of 2.5 nL droplets on the 550.^41^ Comparatively, the DMSO preset on 550 had the highest median fluorescence at all DNA concentrations. This could be due to the difference in surface tension between DMSO and DNA solutions (42.92 mN/m^42^ for DMSO and 71.99 mN/m^43^ for water at 25 °C), which could lead to poor calibration by the Echo fluid dispense algorithm. This mismatch of expected and actual fluid properties may also cause the increased interquartile range using the DMSO preset on the 550 compared to B2 and P2 that were not statistically different from each other. However, B2 showed the lowest day-to-day variability (Figure 3C) when compared to the other settings (Figure S3A,B) and was carried forward as the DNA transfer setting.

### Plate Validation to Assess Material Drift and Edge Effects

We next evaluated plate uniformity using an interleaved signal format assay to identify confounding data artifacts, such as material drift and edge effects, in a plate-based assay (Figure 3D–E).^44^ Using a 1 μL CFE reaction expressing J23119-sfGFP as our pilot assay, we first uniformly dispensed 900 nL of CFE reagents into all wells of a 384-well plate using the Echo 525. Then, we added 20 nM (maximum output), 10 nM (midpoint output), and 5 nM (minimum output) final concentrations of DNA via a 100 nL transfer using the Echo 550 B2 preset into alternating columns (Figure 3D). The data was then graphed first with well number by row and then column on the *x*-axis to identify row-wise drift or edge effects (Figure 3F). Visually, there are no trends within rows. For all signal conditions, the distribution of data is consistent. No rows show calculated drift in a consistent, predominant pattern above 20% of the mean. When graphing the same data with the *x*-axis representing well number by column, then by row, no column-wise drift effects are seen either (Figure 3G). The distribution of data is consistent, and there is no trend of calculated drift above 20% between columns. Therefore, no drift or edge effects are present. Further description of quantitative and qualitative acceptance criteria is explained elsewhere.^44^

It is noted that the variance is higher for the 20 nM DNA condition shown in Figure 3F,G and is greater than the data in Figure 3B. This could be due to day-to-day variability in the Echo or error introduced by Echo dispense patterns. Each condition in Figure 3B was dispensed as a block before moving to the next condition. In contrast, Figure 3F,G was dispensed as alternating rows, which was more demanding for the Echo algorithm.

### Plate-Based Normalization of LETs

To enable high-throughput workflows, LETs can be used in place of plasmid DNA, circumventing the need for laborious and slow plasmid propagation steps.^28,45^ LETs encoding the protein of interest can be amplified by the PCR and added to CFE directly. However, we found that the concentration of aTF DNA in reaction affects biosensor signal, necessitating the normalization of LET DNA concentration (Figure S4). Therefore, we developed a method to rapidly normalize the LET DNA concentration in plates across a DNA library (Figure S5A).

We first amplified the LETs via PCR using a plasmid library as our template. We next showed that we could quantify our LET yields using the commercial QuantiFluor kit for dye-based DNA concentration measurements (Figure S5B). We found that QuantiFluor could robustly measure the LET DNA concentration across all tested buffering conditions: spent PCR buffer, purified LET eluted in water, and a 50/50 mixture of spent PCR buffer and water. The concentrations of LETs measured via QuantiFluor were accurate when compared to Qubit quantification as a standard (Figure S5C). We next investigated the accuracy of the Echo in normalizing aTF LET DNA. To do this, we diluted a pilot library of 35, 50, and 70 ng/μL DNA down to 7.5 ng/μL (10 nM) of DNA. We chose 35 to 70 ng/μL DNA as a representative range of typical PCR yields in our hands. The Echo showed accurate and precise normalization of DNA at all initial concentrations (Figure S5D).

### CFE Workflow for Engineering Transcription Factors

**Figure 4:**
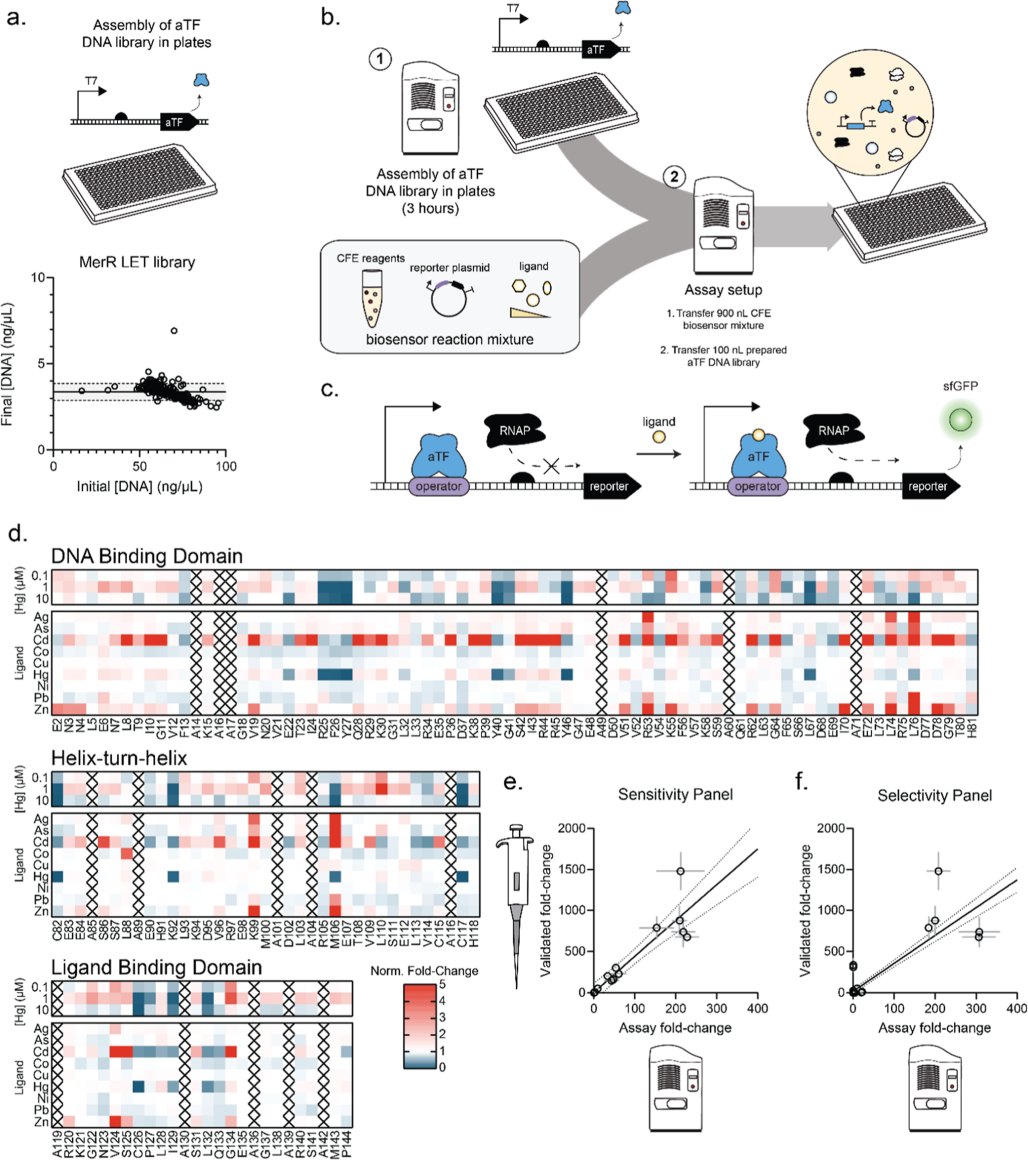
High-throughput cell-free platform for screening transcription factor variants. (A) Schematic of aTF activator mechanism and engineering workflow. MerR activators bind a specific operator site and activate transcription via endogenous RNA polymerase upon ligand binding. (B) Our workflow starts with a semiautomated DNA assembly method for preparing normalized concentrations of aTF variants. Then, 900 nL of CFE biosensor mixture (bulk CFE + ligand of interest) is dispensed using the Echo 525, and 100 nL of the normalized aTF library is dispensed on top. (C) MerR DNA library is prepared and normalized in plates. Alanine scanning mutagenesis libraries consisting of 127 aTFs are prepared according to the workflow described in Figure S5. The initial DNA concentration is displayed on the *x*-axis, and the final DNA concentration is displayed on the *y* axis. Concentrations were determined via QuantiFluor fluorescence. Solid line represents the final average concentration and dotted lines represent one standard deviation from the mean. The shaded region represents the region between one standard deviation above the mean and one standard deviation below the mean. (D) Alanine scanning mutagenesis throughout MerR protein assayed against a panel of ligand conditions for sensitivity (0.1, 1, and 10 μM Hg) and selectivity (Ag, As, Cd, Co, Cu, Hg, Ni, Pb, and Zn). All selectivity ligands were used at 100 μM except for 20 μM zinc and 10 μM mercury. Protein structure is divided into functional domains. Color bar represents variant fold-change normalized to wild type fold-change for the same ligand condition. Each replicate consists of a plate of all MerR variants assayed against all ligand conditions once. Each replicate contains six wild type MerR reactions per ligand condition as controls. Replicates were set up on different days. Data represents normalized fold-change calculated for each day and averaged together. A black X represents a variant that had alanine as wild type residue. (E) Manual validations show correlation with Echo assay for sensitivity ligand panel. Five variants were randomly selected (E2A, I10A, V19A, F26A, and R105A) and assayed against 0, 0.1, 1, and 10 μM Hg alongside wild type. Assay fold-change is graphed on the *x*-axis, with error bar representing standard deviation between single replicate fold-change over 2 days. Hand validated fold-change is graphed on the *y*-axis. Average and standard deviation from technical triplicates were calculated for each condition. Fold-change was then calculated with error bars representing propagated error from dividing average fluorescence in the presence of ligand by average fluorescence for the no ligand condition. Two technical replicates were set up for each reaction. (F) Manual validations show high correlation with Echo assay for selectivity panel of ligands. The same 5 variants and wild type from (B) were assayed against Ag, As, Cd, Co, Cu, Hg, Ni, Pb, Zn, and no ligand by hand and compared to assay fold-change. Assay and hand-validated fold-change and error were calculated the way described in (B).

After establishing parameters for high-throughput, cell-free reaction assembly using the Echo, we decided to apply the workflow as a proof-of-concept to assess the mutability of the MerR transcription factor from *Escherichia coli* due to its relevance as a heavy metal diagnostic.^46^ We subjected MerR to alanine scanning mutagenesis, substituting the inert, nonbulky alanine at each wild type residue for the purpose of discerning protein sequence-function relationships.^47−49^ We then normalized our 127-member MerR library DNA to a target dilution of 4 ng/μL, which was identified as 10 times the optimal aTF DNA concentration for activity (Figure S4). The average MerR LET concentrations post-normalization was 3.36 ± 0.50 ng/μL (Figure 4A). The initial yield of the library was roughly between 50 and 80 ng/μL of DNA. However, the average normalization was 20% below its concentration target, with a slight negative correlation between initial concentration and final concentration. This trend of the Echo transferring volumes lower than anticipated was also seen in the bulk CFE accuracy test (Figure 2A). This could be due to a hardware calibration error or potential maintenance issue, which could be addressed through the generation of a standard curve or fluid transfer optimizations, as described in Figure 3. For this assay, the yield was sufficient for biosensor activity.

We then screened our library for biosensor leak, selectivity, sensitivity, and dynamic range in parallel. MerR is an analyte-binding transcription factor that controls expression of a downstream reporter gene in response to binding mercury (Figure 4C).^50^ The MerR protein sequence specifically binds to its cognate operator site in the absence of a ligand, repressing transcription of a downstream gene. Upon ligand binding, transcription is activated by distorting the bound operator DNA, allowing the RNA polymerase (RNAP) to bind and transcribe. MerR biosensor reactions can therefore be assembled as a two-DNA system by combining DNA encoding a MerR protein with a separate plasmid encoding a downstream reporter gene controlled by an operator sequence. We first transferred 900 nL of a CFE biosensor reaction mixture containing CFE reagents, pMer-sfGFP reporter plasmid, and the ligand of interest into 384-well microplates using the Echo (Figure 4B). Next, we added 100 nL of each aTF LET into unique wells, generating hundreds of biosensor reactions with unique aTF/ligand pairs, and measured sfGFP in each CFE biosensor reaction as a readout for MerR activity.

To assay for sensitivity, the entire 127-member aTF library was screened against 0, 0.1, 1, and 10 μM mercury, creating 508 CFE reactions (Figure 4D). Wild type MerR is sensitive to mercury at 1 μM (Figure S6A). The dynamic range (defined as fold-change) was calculated for each replicate by dividing the measured reaction fluorescence for each replicate in the presence of ligand by the baseline fluorescence (i.e., leak) in the absence of ligand, normalized to the wild type fold-change for the same ligand condition. We were able to identify the three cysteines responsible for ligand binding:^50−52^ C82, C117, and C126 through their loss of activity at all concentrations of mercury. Furthermore, E22A in the DBD and P127A in the LBD showed a loss of activity compared with the wild type. This is supported by previous studies, in which an E22K^51^ mutant and P127L^52^ mutant were both found to cause loss of function phenotypes, with P127 being hypothesized to play a major role in metal binding.^53^ Because we comprehensively mapped sequence to function across the entire MerR protein, we also observed amino acid positions that impacted activity, which have not been previously reported.

To assay for selectivity, the aTF library was screened against ligands known to interact with MerR-family transcription factors: silver, arsenic, cadmium, cobalt, copper, mercury, nickel, lead, zinc, as well as no ligand (1270 unique reactions).^46^ Wild type MerR shows promiscuity for none of the tested ligands (Figure S6A). The library shows a broad preference for promiscuity toward cadmium and zinc compared to wild type. This is also supported by literature precedent, with multiple studies aimed at engineering cadmium selectivity in MerR.^53,54^ In both of those works, the K99 and M106 residues were prominent sites for engineering cadmium promiscuity. In this study, mutants K99A and M106A both displayed increased promiscuity for cadmium, while M106A also showed a major loss of mercury-dependent activation. In our assay, K99A and M106A displayed a strong dynamic range for multiple ligands in our screen, suggesting these residues as potential sites for engineering promiscuity for zinc, silver, and arsenic, as well. One work performed random mutagenesis throughout all 144 codons of MerR and selected for cadmium promiscuity.^54^ The residues R53, E72, L74, L76, S125, and S131 were also identified in our screen as sites for engineering cadmium promiscuity, encompassing all of the single point mutants identified in a selection study aimed at investigating MerR selectivity for cadmium through random protein variants.^54^

We next randomly selected 5 variants (E2A, I10A, V19A, F26A, and R105A) from our MerR library to manually validate against both the sensitivity (Figure 4E) and selectivity ligand panels alongside wild type (Figure 4F). Both reaction sets showed a correlation in relative fold-change across a range of activities. The fold-change magnitude for the MerR assay was lower than the 10 μL manual reactions as expected, likely due to the lower reaction volume limiting the signal range.

To demonstrate the robustness of our workflow, we then screened an additional aTF, namely, the cadmium-sensing CadR transcription factor with a 134-member alanine scan library (Figure S7A). Wild type CadR shows activity beginning at 10 μM Cd and promiscuity for most of the tested selectivity panel ligands (Figure S6B). CadR overall had a greater abundance of negative mutants from both the sensitivity and selectivity panel compared to MerR. CadR has two distinct metal binding sites consisting of C77, C112, C119, and N81 forming the first, and H87, H90, E62, H140, and H145 forming the second.^55^ Of these, C112A, C119A, H87A, and H90A all show major loss of function phenotypes in both the sensitivity and selectivity panels. The rest of the metal binding residues either display moderate loss of function or, in the case of N81A, potential promiscuity increases. Modeling software could be used to further investigate these potential structural and interaction effects.^56,57^ Despite having greater wild type promiscuity, the CadR library showed far fewer promiscuity shifts than the MerR library, suggesting that the structure is less mutable. This could be due to the coordination of two ligand binding sites, compared to MerR only having one ligand binding site. As with MerR, we manually validated 5 random variants from our CadR library for sensitivity and selectivity panels alongside wild type (Figure S7B,C) and found strong correlations in relative fold-change across both sensitivity and selectivity.

## Conclusions

We developed a high-throughput CFE workflow for transcription factor engineering that leveraged liquid handling robots. A key feature was the identification of critical assay parameters for protocol validation with suggested pilot screens to evaluate confidence and consistency. With this workflow, assays with similar precision can be tailored to specific applications.

We also provided a framework for optimized final miniaturized reaction formats and unique reagent transfers and demonstrate their efficacy for engineering biosensors. For sensitive systems such as this one with multiple DNA inputs, we additionally created a DNA preparation workflow that allows the precise dilution of DNA to a single concentration in plates that takes <3 h. We applied our workflow to engineer the aTFs MerR and CadR using an alanine mutagenesis screen. The CFE workflow enabled the combinatorial setup of 3682 unique CFE biosensor reactions in 48 h by a single operator. This assay demonstrated a strong ability to identify changes in complex characteristics such as dynamic range, promiscuity, and limit of detection. The data was confirmed through literature precedent and a strong linear correlation with hand-validated dynamic range at the 10 μL format.

A limitation of this work compared to traditional selection strategies is that the overall aTF library size is orders of magnitude smaller than what can be accessed by directed evolution or droplet sorting techniques. To increase scale, 1536-well plates could be used with smaller volume sizes (<1 μL). Evaporation risks could be reduced through this plate geometry; however, further efforts to offset evaporation, such as humidification of the reaction throughout set up and incubation may be needed. Future works could leverage this platform to coengineer unique aTF, ligand, and reporters in parallel. With the data consistency demonstrated here, the effect of all three variables could be compared directly, investigating interactions between amino acid/DNA motif pairs and how they affect biosensor performance characteristics such as limit of detection, specificity, and dynamic range.

Overall, we have provided an automated, CFE method for screening multi-component cell-free systems, such as transcription factors, for multiple characteristics at once. We anticipate that this approach can be used for a broad range of synthetic biology projects. Integration of CFE workflows with machine learning models will further accelerate biological design.

## Methods

### DNA Assembly and Purification

All plasmids used in this study, apart from the MerR DNA library, were from Addgene. A list of all plasmids, including descriptions and Addgene accession IDs, is presented in Table 1. The MerR DNA library and CadR DNA library templates were synthesized by Twist Biosciences as plasmids in a pJL1 backbone as variants of pT7MerR (Addgene ID 167213) and pT7 CadR (Addgene ID 167217), respectively. LETs of each MerR variant were produced using a forward (CGATAAGTCGTGTCTTACCG) and reverse (gcataagcttttgccattctc) primer pair that bound to the PJL1 backbone. pT7-MerR plasmid and library DNA variants were used at a final concentration of 0.5 nM DNA in reaction. All other plasmids in this study were used at a 20 nM concentration in reaction.

**Table 1:** Summary of Plasmids Used in This Manuscript^a^

plasmid description	addgene ID
J23119-pHP14-sfGFP	136942
pT7-MerR	167213
pT7-CadR	167217
pMer-sfGFP	167220
pCad-sfGFP	167221

a pT7 refers to the wild type T7 promoter TAATACGACTCACATATA. J23119 is the consensus *E. coli* RNAP promoter. All plasmids are on Addgene.

### Cell Extract Preparation

Cell extract was prepared as previously described for expression of endogenous transcriptional machinery.^58^ Briefly, BL21 Star (DE3) (Thermo Fisher Scientific C601003) was grown in 2X YT + P media (16 g/L tryptone, 10 g/L yeast extract, 5 g/L sodium chloride, 7 g/L potassium phosphate dibasic, and 3 g/L potassium phosphate monobasic) adjusted to pH 7.2. The strain is grown to an optical density (OD_600_) of 0.5 at 37 °C shaking at 250 rpm and induced with isopropyl ß-D-1-thiogalactopyranoside (IPTG) to a final concentration of 100 μM. The culture is then grown to an OD_600_ of 3.0. The cells are then pelleted by a 15 min spin at 5000*g* at 4 °C and washed with 25 mL of wash buffer (14 mM magnesium glutamate, 60 mM potassium glutamate, 10 mM of Tris base, brought to pH 7.8) three times. Cells are then lysed by passing through an Avestin EmulsiFlex-B15 homogenizer at 24,000 ψ. Cell debris is pelleted through a 10 min spin at 12,000*g* at 4 °C and the clarified lysate is incubated at 37 °C with shaking at 250 rpm for 1 h. Lysate is spun again at 12,000*g* for 10 min at 4 °C then dialyzed using a 10K MWCO dialysis membrane in dialysis buffer (14 mM magnesium glutamate, 60 mM potassium glutamate, 5 mM Tris base, 1 mM DTT, brought to pH 8.0) for 3 h at 4 °C. After dialysis, the lysate is centrifuged at 12,000*g* for 10 min at 4 °C, and the supernatant is flash-frozen on liquid nitrogen and stored at −80 °C.

### CFE Reaction

CFE reactions were constructed as previously described.^58^ Reaction compositions were developed and reported before^7,59,60^ In brief, they were composed of 8 mM magnesium glutamate, 10 mM ammonium glutamate, 130 mM potassium glutamate, 1.2 mM ATP, 0.5 mM of CTP, GTP, and UTP, respectively, 0.17 mg/mL of *E. coli* MRE600 tRNA (Roche 10109541001), 100 mM NAD, 50 mM CoA, 5 mM oxalic acid, 1 mM spermidine, 1 mM putrescine, 57 mM HEPES at a pH of 7.2, 33.3 mM PEP, 2 mM of each amino acid, and 20% v/v *E. coli* extract described above. DNA and water constitute the remainder of the reaction. By hand, manual reactions were set up as 10 μL reactions in 384-well clear bottom plates (Corning 3712). Echo-assembled reactions were set up in 384-well V-bottom plates (Bio Rad HSP3805). Reactions were incubated at 30 °C for 15 h and read on the BioTek Synergy H1 plate reader with 485 and 528 wavelengths for excitation and emission, respectively. Fluorescence was quantified by fluorescein isothiocyanate (FITC) (Sigma-Aldrich 46950). Standard curves were assembled by diluting FITC in 50 mM sodium borate at pH 8.5.

### Echo-Assisted Assembly of Cell-Free Expression Reactions

Two Echo Acoustic Liquid Handlers were used in this study, the 525 (LabCyte 001–10080) and 550 (Labcyte 001–2000). While not used in this study, Echo 550 has been replaced by the 650 series (Beckman Coulter 001–16079). The Echo dispense data from Figure 2 and all subsequent 900 nL transfers of bulk CFE reagents were done using an Echo Qualified 384-Well Polypropylene 2.0 Plus Microplate on the BP setting (LabCyte PP-0200). For the normalization of aTF DNA in the form of LETs, LET DNA was transferred from the Echo Qualified 384-well cyclic olefin copolymer LDV Microplate on the 525 (Beckman Coulter 001–13070). For the 100 nL transfer of aTF LETs, the Echo Qualified 384-Well LDV Microplate plate on 550 nL (LabCyte LP-0200) was used. Reactions were programmed on the Echo using either Plate Reformat or CherryPick software.

### Data Analysis and Statistics

Replicate numbers are described in the associated figure legends. Generally, reactions set up by hand at 10 μL had at least 2 technical replicates per reaction, and individual data points were plotted. Graphs were generated using the GraphPad Prism 9 software, and statistical methods were also performed using built-in GraphPad Prism 9 software as well.

## Data Availability

Source data for all figures will be available upon request.

## Supporting Information Available

Normalized fold-change for the MerR and CadR library sensitivity and selectivity assays (XLSX)
PCR buffer tolerance for CFE reactions, J23119-sfGFP DNA titration, fluid type optimizations, sensor and reporter DNA titrations, DNA normalization workflow, and CadR mutagenesis assay and validation (PDF)
