# Supplementary material for: An Automated Cell-Free Workflow for Transcription Factor Engineering
Source: ACS Synth Biol. 2024 Oct 7;13(10):3389–99. doi: 10.1021/acssynbio.4c00471 (PMC11494693; doi:10.1021/acssynbio.4c00471)
Supplement: Supplementary file 2 — sb4c00471_si_002.pdf [file sb4c00471_si_002.pdf]

Supplementary Materials for:

An automated cell-free workflow for transcription factor engineering

Holly M. Ekas[a,b,c], Brenda Wang[a,b,c], Adam D. Silverman[a,b,c], Julius B. Lucks[a,b,c,d], Ashty S. Karim[a,b,c], Michael C. Jewett[a,b,c,e,f,g*]

Affiliations:

a. Department of Chemical and Biological Engineering, Northwestern University, Evanston, IL 60208, USA
b. Chemistry of Life Processes Institute, Northwestern University, Evanston, IL 60208, USA
c. Center for Synthetic Biology, Northwestern University, Evanston, IL 60208, USA
d. Center for Engineering Sustainability and Resilience, Northwestern University, Evanston, IL, 60208, USA
e. Robert H. Lurie Comprehensive Cancer Center, Northwestern University, Chicago, IL 60611, USA
f. Simpson Querrey Institute, Northwestern University, Chicago, IL 60611, USA
g. Department of Bioengineering, Stanford University, Stanford, CA 94305

* Corresponding author

Correspondence

Michael C. Jewett, mjewett@stanford.edu

This PDF file includes:

Supplementary Figures 1 to 7

Other Supplementary Materials for this manuscript include the following:

Supplementary Data included as separate files.

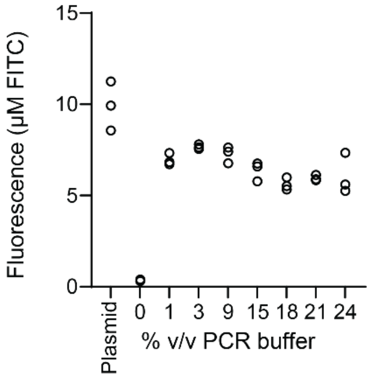

Supplemental Figure 1. PCR buffer tolerance in cell-free biosensor reactions. MerR LET-based biosensor is tolerant to spent PCR buffer. MerR was expressed via LET, with a plasmid control, and spent PCR buffer was titrated in. MerR shows high tolerance to PCR buffer up to ~10%, with minimal variations to overall sensor activity. Three technical replicates were set up by hand in 10 µL volumes. Individual replicates are plotted.

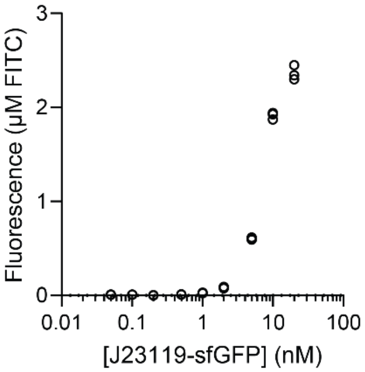

Supplemental Figure 2. J23119-sfGFP DNA titration. Titration of J23119-sfGFP DNA shows linear relationship between DNA concentration and reaction fluorescence between 2 nM and 20 nM DNA. Three technical replicates were set up by hand in 10 µL volumes. Individual replicates are plotted.

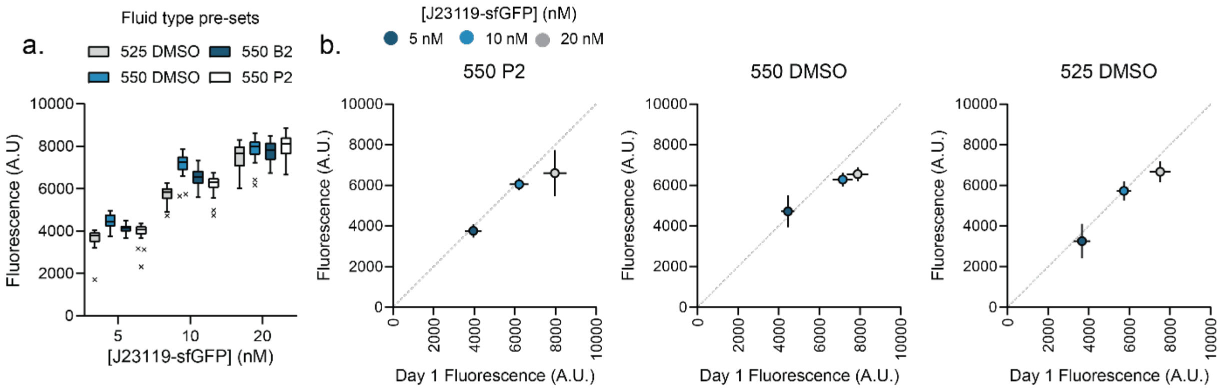

Supplemental Figure 3. Fluid type optimizations for additional Echo settings. (**A**) Day 2 data for assessing the precision of fluid type pre-sets for transferring 100 nL of DNA into a 1 µL reaction on both the 550 and 525. The Echo dispensed three different DNA concentrations to achieve 5, 10, and 20 nM of J23119-sfGFP in CFPS. 32 technical replicates were set up per concentration/pre-set using the Echo. Graphed with Tukey method. (**B**) Data from Figure 3B and Supplemental Figure S1D graphed together for each fluid pre-set dispensing 100 nL of DNA. Points represent average and error bars show standard deviation. Black line is simple linear regression with Equation shown and R^2 shown on graph. Dotted gray line is line of identity.

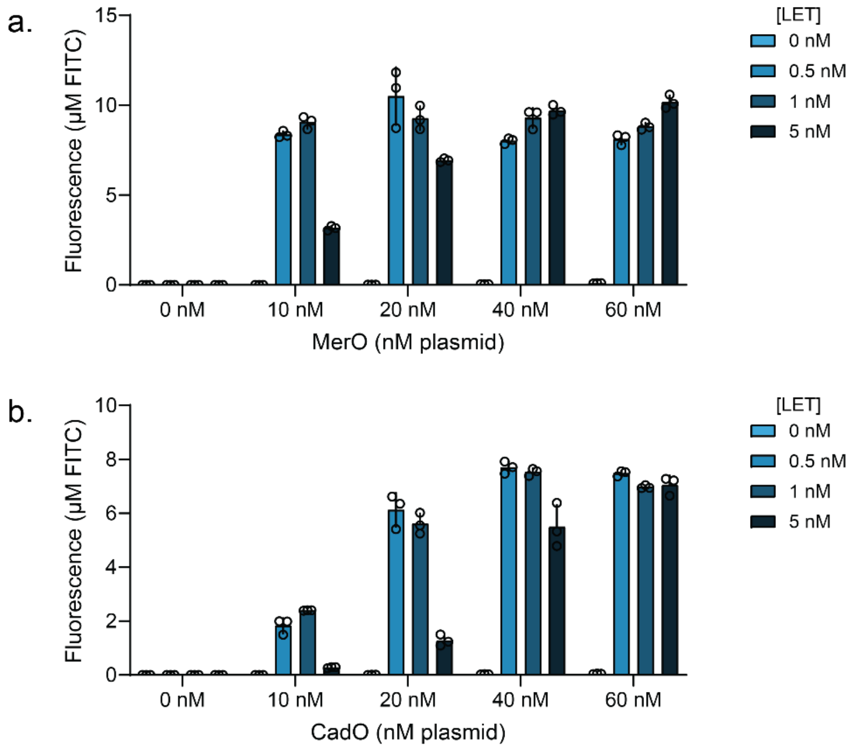

Supplemental Figure 4. Sensor and reporter DNA titration for MerR and CadR. (**A**) Co-titration of aTF DNA (LET) and reporter DNA (plasmid) show biosensor activity is dependent on DNA concentration for MerR. Technical replicates were set up by hand in 10 μL volumes. Individual replicates are plotted, bars represent average and error bars represent standard deviation. (**B**) Co-titration of aTF DNA (LET) and reporter DNA (plasmid) show biosensor activity is dependent on DNA concentration for CadR. Technical replicates were set up by hand in 10 μL volumes. Individual replicates are plotted, bars represent average and error bars represent standard deviation.

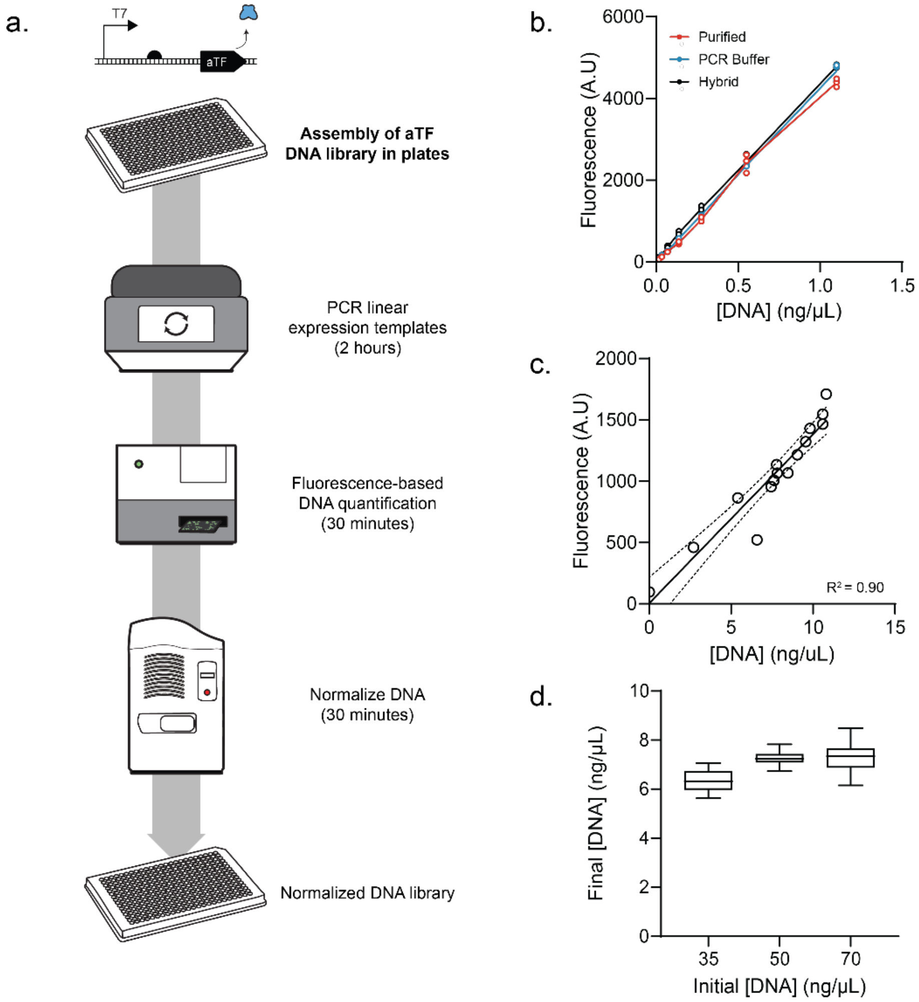

Supplemental Figure 5. DNA normalization workflow experiments. (**A**) Schematic semi-automated aTF DNA library preparation workflow. From a small amount of library plasmid DNA, linear expression templates are amplified via PCR. These templates are then quantified via plate-based fluorescent dyes. The Echo dispenses a combination of both water and aTF for each aTF according to concentration to normalize the DNA concentration. This workflow can be completed in under 3 hours. (**B**) QuantiFluor DNA quantification shows no change in fluorescence for different buffering conditions. Purified LETs are eluted in water, PCR buffer are LETs spiked into spent PCR buffer, and hybrid is LETs spiked into a 50/50 mixture of spent PCR buffer and water. Three replicates were set up by hand according to manufacturer's instructions. Individual replicates are shown on the graph. (**C**) QuantiFluor fluorescence correlates strongly with Qubit standard. LETs are quantified both through Qubit (x-axis) and QuantiFluor (y-axis). Solid line is simple linear regression with associated R^2 shown on the graph and dotted lines are the 95% confidence interval. 16 LETs were quantified. (**D**) Echo 550 with the LDV B2 setting is sufficient for dispensing varying amounts of DNA at 35, 50, and 70 ng/µL starting DNA to a single final concentration. Target final concentration was 7 ng/µL. Graphed with Tukey method. 16 replicates were set up for each DNA concentration.

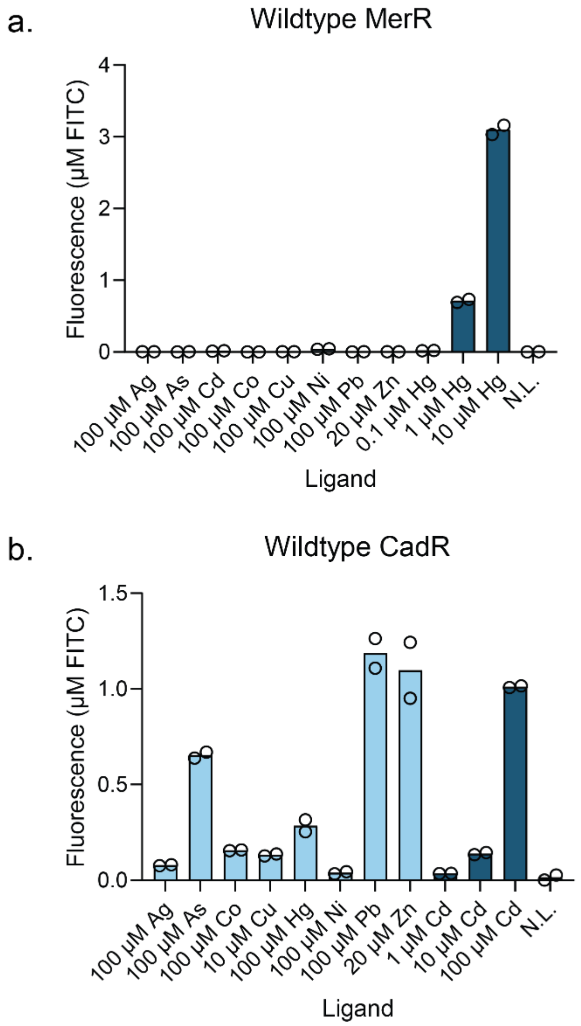

Supplemental Figure 6. Wildtype MerR and CadR sensitivity and selectivity in cell-free reactions. (**A**) Wildtype MerR selectivity and sensitivity. MerR LETs were assayed for selectivity (Ag, As, Co, Cu, Hg, Ni, Pb, Zn), sensitivity (0.1, 1, and 10 µM Hg), and with no ligand using 0.5 nM LET DNA with 20 nM of reporter plasmid in final CFE reaction. All selectivity ligands were used at 100 µM except for 20 µM zinc and 10 µM mercury. Graphs show individual replicates plotted individually, with bars representing the average. (**B**) Wildtype CadR selectivity and sensitivity. CadR LETs were assayed for selectivity (Ag, As, Co, Cu, Hg, Ni, Pb, Zn), sensitivity (1, 10, 100 µM Cd), and with no ligand using 0.5 nM LET DNA with 20 nM of reporter plasmid in final CFE reaction. All selectivity ligands were used at 100 µM except for 20 µM zinc and 10 µM mercury. Graphs show individual replicates plotted individually, with bars representing the average.

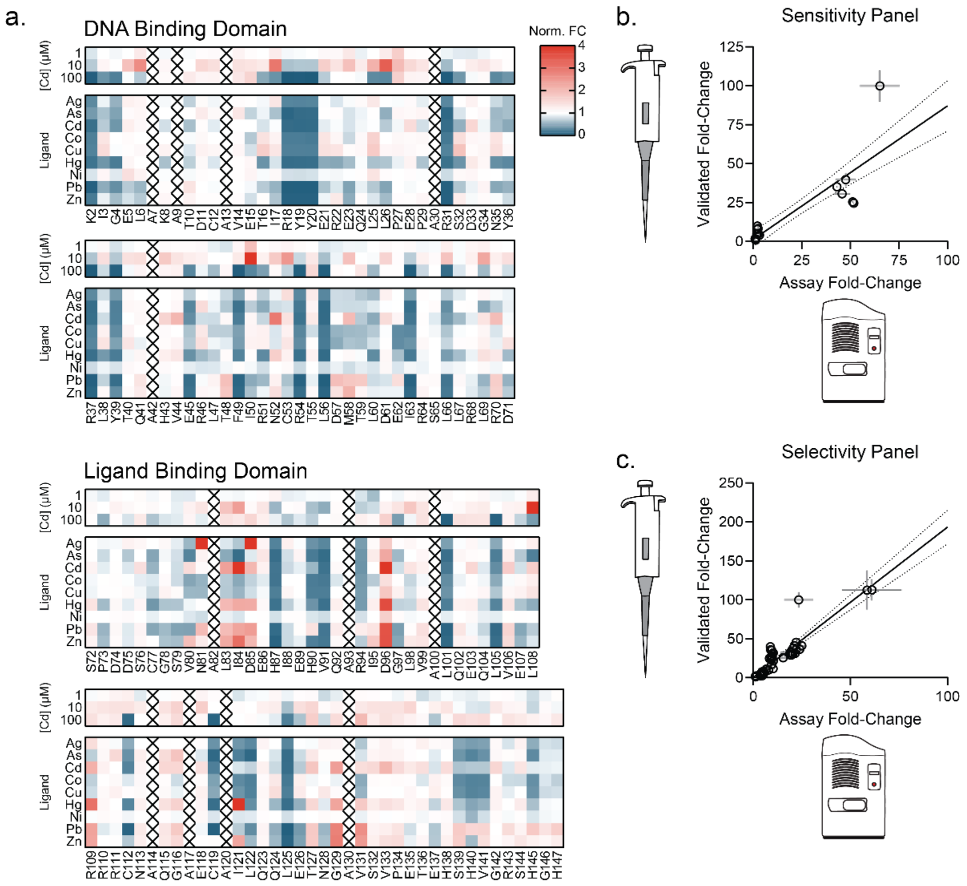

Supplemental Figure 7. High-throughput cell-free screening of CadR variants for sensitivity and selectivity. (**A**) Alanine scanning mutagenesis throughout CadR protein assayed against a panel of ligand conditions for sensitivity (1, 10, 100 µM Cd) and selectivity (Ag, As, Cd, Co, Cu, Hg, Ni, Pb, Zn). All selectivity ligands were used at 100 µM except for 20 µM zinc and 10 µM mercury. Protein structure is divided into functional domains. Color bar represents variant fold-change normalized to wild type fold-change for the same ligand condition. Each replicate consists of a plate of all CadR variants assayed against all ligand conditions once. Each replicate contains six wildtype CadR reactions per ligand condition as controls. Replicates were set up on different days. Data represents normalized fold-change calculated for each day and averaged together. A black X represents a variant that had alanine as wild type residue. (**B**) Manual validations show high correlation with Echo assay for sensitivity ligand panel. 5 variants were randomly selected (C12A, G34A, H43A, D74A, G129A) and assayed against 0, 1, 10, and 100 µM Cd alongside wild type. Assay fold-change is graphed on x-axis, with error bar representing standard deviation between single replicate fold-change over two days. Hand validated fold-change is graphed on y-axis. Average and standard deviation from technical triplicates were calculated for each condition. Fold-change was then calculated with error bars representing propagated error from dividing average fluorescence in the presence of ligand by average fluorescence for the no ligand condition. Two technical replicates were set up for each reaction. (**C**) Manual validations show high correlation with Echo assay for selectivity panel of ligands. The same 5 variants and wild type from (B) were assayed against Ag, As, Cd, Co, Cu, Hg, Ni, Pb, Zn, and no ligand by hand and compared to assay fold-change. Assay and hand validated fold-change and error were calculated as described in (B).
